# NUP98‐BPTF promotes oncogenic transformation through PIM1 upregulation

**DOI:** 10.1002/cam4.7445

**Published:** 2024-06-28

**Authors:** Mina Noura, Sakura Tomita, Takahiko Yasuda, Shinobu Tsuzuki, Hitoshi Kiyoi, Fumihiko Hayakawa

**Affiliations:** ^1^ Division of Cellular and Genetic Sciences, Department of Integrated Health Sciences Nagoya University Graduate School of Medicine Nagoya Japan; ^2^ Clinical Research Center, National Hospital Organization Nagoya Medical Center Nagoya Japan; ^3^ Department of Biochemistry Aichi Medical University School of Medicine Nagakute Japan; ^4^ Department of Hematology and Oncology Nagoya University Graduate School of Medicine Nagoya Japan

**Keywords:** cell transformation, leukemia, NUP98‐BPTF, PIM1, RNA‐seq

## Abstract

**Introduction:**

Nucleoporin 98 (NUP98) fusion proteins are recurrently found in leukemia and are associated with unfavorable clinical outcomes. They are distributed to the nucleus and contribute to leukemogenesis via aberrant transcriptional regulation. We previously identified NUP98‐BPTF (NB) fusion in patients with T‐cell acute lymphoblastic leukemia (T‐ALL) using next‐generation sequencing. The FG‐repeat of NUP98 and the PHD finger and bromodomain of bromodomain PHD finger transcription factor (BPTF) are retained in the fusion. Like other NUP98 fusion proteins, NB is considered to regulate genes that are essential for leukemogenesis. However, its target genes or pathways remain unknown.

**Materials and Methods:**

To investigate the potential oncogenic properties of the NB fusion protein, we lentivirally transduced a doxycycline‐inducible NB expression vector into mouse NIH3T3 fibroblasts and human Jurkat T‐ALL cells.

**Results:**

NB promoted the transformation of mouse NIH3T3 fibroblasts by upregulating the proto‐oncogene *Pim1*, which encodes a serine/threonine kinase. NB transcriptionally regulated *Pim1* expression by binding to its promoter and activated MYC and mTORC1 signaling. PIM1 knockdown or pharmacological inhibition of mTORC1 signaling suppressed NB‐induced NIH3T3 cell transformation. Furthermore, NB enhanced the survival of human Jurkat T‐ALL cells by inactivating the pro‐apoptotic protein BCL2‐associated agonist of cell death (BAD).

**Conclusion:**

We demonstrated the pivotal role of NB in cell transformation and survival and identified PIM1as a key downstream target of NB. These findings propose a promising therapeutic strategy for patients with NB fusion‐positive leukemia.

## 1 INTRODUCTION

Translocations involving chromosome 11p15 create the Nucleoporin 98 (*NUP98*) fusion genes in patients with a wide range of hematopoietic malignancies, most notably acute myeloid leukemia (AML) and T‐cell acute lymphoblastic leukemia (T‐ALL).^1^, ^2^ *NUP98* fusion genes are recurrently found in younger patients under 20 years of age and are associated with poor prognosis.^1^, ^3^ Due to the proximity of *NUP98* to the telomere, *NUP98* translocations are often cytogenetically cryptic and undetectable by conventional karyotyping.^2^, ^4^ Recent advances in next‐generation sequencing have led to more frequent detection of *NUP98* fusion genes and the identification of more than 30 different partner genes.^1^, ^2^

The wild‐type NUP98 protein is located on the nucleoplasmic side of the nuclear pore complex (NPC) and regulates the transport of macromolecules between the nucleus and cytoplasm.^5^ NUP98 also has off‐pore functions such as regulation of gene expression^6^, ^7^ and maintenance of transcriptional memory.^8^ In contrast, NUP98 fusion proteins, such as NUP98‐HOXA9, NUP98‐HOXD13, and NUP98‐NSD1, are localized to the nucleus^9^, ^10^, ^11^ and regulate the transcription of multiple genes through their FG‐repeat domains, which interact with histone‐modifying enzymes, including CBP/p300 and HDAC1.^12^, ^13^ In addition, most NUP98 fusion proteins form nuclear puncta through liquid–liquid phase separation, which leads to aberrant transcriptional condensates and leukemic transformation.^14^, ^15^ Because different NUP98 fusion proteins activate the transcription of common target genes, including several members of the *HOXA* cluster, *MEIS1*, and *CDK6*, the NUP98 moiety is thought to be important for the binding of NUP98 fusion proteins to target promoters.^9^, ^16^

*NUP98‐BPTF* (*NB*) was first identified in a young adult with T‐ALL by our group using next‐generation sequencing.^17^ To date, NB has also been reported in AML^4^, ^18^ and acute megakaryoblastic leukemia,^19^ indicating that NB occurs in both myeloid and T‐lymphoid leukemias. The *NB* fusion gene encodes a chimeric protein that juxtaposes the FG‐repeat domain of NUP98 to the carboxy‐terminal portion of the bromodomain PHD finger transcription factor (BPTF), a core subunit of the nucleosome remodeling factor. The PHD finger of BPTF specifically binds to the trimethylation of histone H3 lysine 4 (H3K4me3), which is associated with the transcription start sites of active genes.^20^, ^21^ BPTF regulates chromatin accessibility and facilitates the transcriptional activation of target genes.^21^, ^22^ Given that the NUP98 moiety is involved in binding to target promoters, it is hypothesized that the PHD finger of BPTF promotes transcriptional activation by regulating chromatin structure. However, the essential genes or pathways that are targeted by NB remain unclear. Here, we investigated the cell‐transforming ability of NB and explored the target genes regulated by NB. Our study identified the proto‐oncogene *PIM1*, which is indispensable for NB‐mediated oncogenic transformation, as a key downstream target of NB.

## 2 MATERIALS AND METHODS

### 2.1 Cell lines

Human embryonic kidney (HEK) 293 T cells and mouse NIH3T3 fibroblasts were obtained from American Type Culture Collection (ATCC, USA) and maintained in Dulbecco's modified Eagle's medium (Wako, Japan) supplemented with 10% fetal bovine serum (FBS; Thermo Fisher Scientific, USA) and 1% penicillin–streptomycin (PS; Wako) under 5% CO_2_ and 95% air at 37°C. Human Jurkat T‐ALL cells were cultured in Roswell Park Memorial Institute 1640 medium (Wako) containing 1% PS in a humidified incubator with 5% CO_2_ and 95% air at 37°C.

### 2.2 Reagents

Rapamycin and TP‐3654 were purchased from MedChemExpress, USA.

### 2.3 Expression plasmids

Human *NB* cDNAs were amplified by overlapping PCR using the cDNA of *NUP98* and *BPTF* as templates and then inserted into pCSII‐Tet on IRES‐GFP.^23^ All the PCR products were verified by DNA sequencing.

### 2.4 Specific short hairpin RNAs (shRNAs) interference

shRNAs targeting mouse *Pim1* were designed and sub‐cloned into pENTR4‐H1tetOx1 and CS‐RfA‐ETR vectors. These vectors were kindly provided by Dr. H. Miyoshi (RIKEN BRC, Japan). The target sequence for mouse *Pim1* shRNA was 5′‐TGCAAGACCTCTTCGACTTTA−3′.

### 2.5 Lentivirus production and transduction

HEK293T cells were transiently co‐transfected with lentiviral vectors, psPAX2, and pMD2.G using PEI Max (MW 40,000) (Polysciences, USA). Then, 48 h after transfection, viral supernatants were collected and immediately used for infection. Successfully transduced cells were sorted using the Aria II flow cytometer.

### 2.6 Immunoblotting

The cells were washed in PBS and then lysed in RIPA buffer (Wako). After centrifugation, the protein content of the supernatants was measured using DC Protein Assay (Bio‐Rad Laboratories, USA). Equal amounts of whole‐cell lysates were separated by SDS/PAGE and then electrotransferred onto polyvinylidene difluoride membranes. The membranes were probed with the following primary antibodies: anti‐GAPDH (0411; Santa Cruz Biotechnology, USA), and anti‐NUP98 (#2288; Cell Signaling Technology, USA), anti‐PIM1(12H8; Santa Cruz Biotechnology), anti‐c‐Myc (#5605; Cell Signaling Technology), anti‐Phospho‐c‐Myc (#13748; Cell Signaling Technology), anti‐p70 S6 Kinase (#34475; Cell Signaling Technology), anti‐Phospho‐p70 S6 Kinase (Thr389) (#9234; Cell Signaling Technology), anti‐BAD (#9239; Cell Signaling Technology), and anti‐Phospho‐Bad (Ser112) (#5284; Cell Signaling Technology). Horseradish peroxidase (HRP)‐conjugated anti‐rabbit IgG and anti‐mouse IgG (Cell Signaling Technology) were used as secondary antibodies. Blots were visualized using ECL Prime Western Blotting Detection Reagent (Cytiva, Japan) and Light‐Capture II (ATTO, Japan) according to the manufacturer's recommendations.

### 2.7 Focus formation assay

NIH3T3 cells were lentivirally transduced with a Doxycycline (Dox)‐inducible NB expression vector and seeded at a density of 1 × 10^6^ cells per 10 cm culture dish. Cells were cultured with or without Dox for 3 weeks. Medium and Dox were changed every other day. After 3 weeks of culture, macroscopic pictures were obtained after staining with 0.5% crystal violet in 20% methanol. In addition, cells were trypsinized and counted using the trypan blue exclusion method.

### 2.8 Immunofluorescence

NIH3T3 cells were lentivirally transduced with a Dox‐inducible NB expression vector, seeded in an 8‐well chamber slide (Thermo Fisher Scientific), and treated with or without Dox for 48 h to induce NB expression. Cells were washed in PBS, fixed with 4% paraformaldehyde, and permeabilized with 0.2% Triton X‐100 in PBS for 10 min. Fixed cells were blocked with Blocking One Histo (Nacalai Tesque, Japan) for 1 h, and then probed with an anti‐NUP98 antibody (#2288; Cell Signaling Technology) at 4°C overnight, followed by incubation with Alexa Fluor 568‐conjugated goat anti‐rabbit secondary antibody (Thermo Fisher Scientific) for 1 h. The cells were washed with PBS, stained with DAPI (Nacalai Tesque), and mounted with ProLong Glass Antifade Mountant (Thermo Fisher Scientific). Images were captured using a laser confocal microscope (LSM880; Carl Zeiss, Germany).

### 2.9 RNA‐seq

RNA‐seq was carried out as previously described.^17^

### 2.10 Gene set enrichment analysis

Gene set enrichment analysis was performed using the Hallmark gene sets from the Molecular Signature Database version 2023.1.Hs. The genes were ranked according to Wald statistics based on the results of DESeq. Table S1 lists all gene sets with FDR <0.25 and nominal *p*<0.05.

### 2.11 Reverse transcription‐quantitative polymerase chain reaction (RT‐qPCR)

Total RNA was isolated using the ReliaPrep RNA Cell Miniprep System (Promega, USA) and reverse‐transcribed using a reverse‐script kit (TOYOBO, Japan) to generate cDNA. RT‐qPCR was performed on Thermal Cycler Dice Real‐Time System II (Takara Bio, Japan) according to the manufacturer's recommendations. The results were normalized to the expression levels of *glyceraldehyde‐3‐phosphate dehydrogenase* (*GAPDH*). The relative expression levels were calculated using the 2^−ΔΔCt^ method.^24^ The primer sequences for RT‐qPCR were *Pim1*‐F GATCATCAAGGGCCAAGTGT; *Pim1*‐R GATGGTTCCGGATTTCTTCA; *Gapdh*‐F ATGACATCAAGAAGGTGGTGAAG; *Gapdh*‐R, TCCTTGGAGGCCATGTAGG.

### 2.12 Chromatin immunoprecipitation‐quantitative polymerase chain reaction (ChIP‐PCR)

The ChIP assay was performed as described in a previous study.^25^ The primer sequences for PCR were F 5′‐CCTCAGTCGTCCTCCGACTC‐3′ and R 5′‐GAGCATCCCCACCTCCAG−3′.

### 2.13 Apoptosis assay

The cells were washed in PBS, suspended in annexin V binding buffer, and then mixed with 5 μL of annexin V and 7‐AAD (BioLegend, USA). The reaction mixtures were incubated for 15 min. After incubation, the cells were diluted, and processed for flow cytometric analysis.

### 2.14 GI_50_ evaluation

Cell viability was assessed by the WST assay using a Cell Counting Kit‐8 (Dojindo, Japan) and ARVO MX (PerkinElmer, USA). Percentage inhibition curves were drawn, and the GI_50_ values of the compounds were calculated based on the median‐effect method.^26^

### 2.15 Statistical analysis

Differences between the control and experimental groups were assessed by a two‐tailed unpaired Student's *t*‐test and declared significant if the *p‐*value was less than 0.05. The equality of variances in two populations was calculated using the *F*‐test. The results are presented as the mean ± standard error of the mean (SEM) of the values obtained from three independent experiments.

### 2.16 Study approval

We did not perform any experiments involving humans or animals in this study.

## 3 RESULTS

### 3.1 NB conferred a transformed phenotype to NIH3T3 cells

**FIGURE 1:**
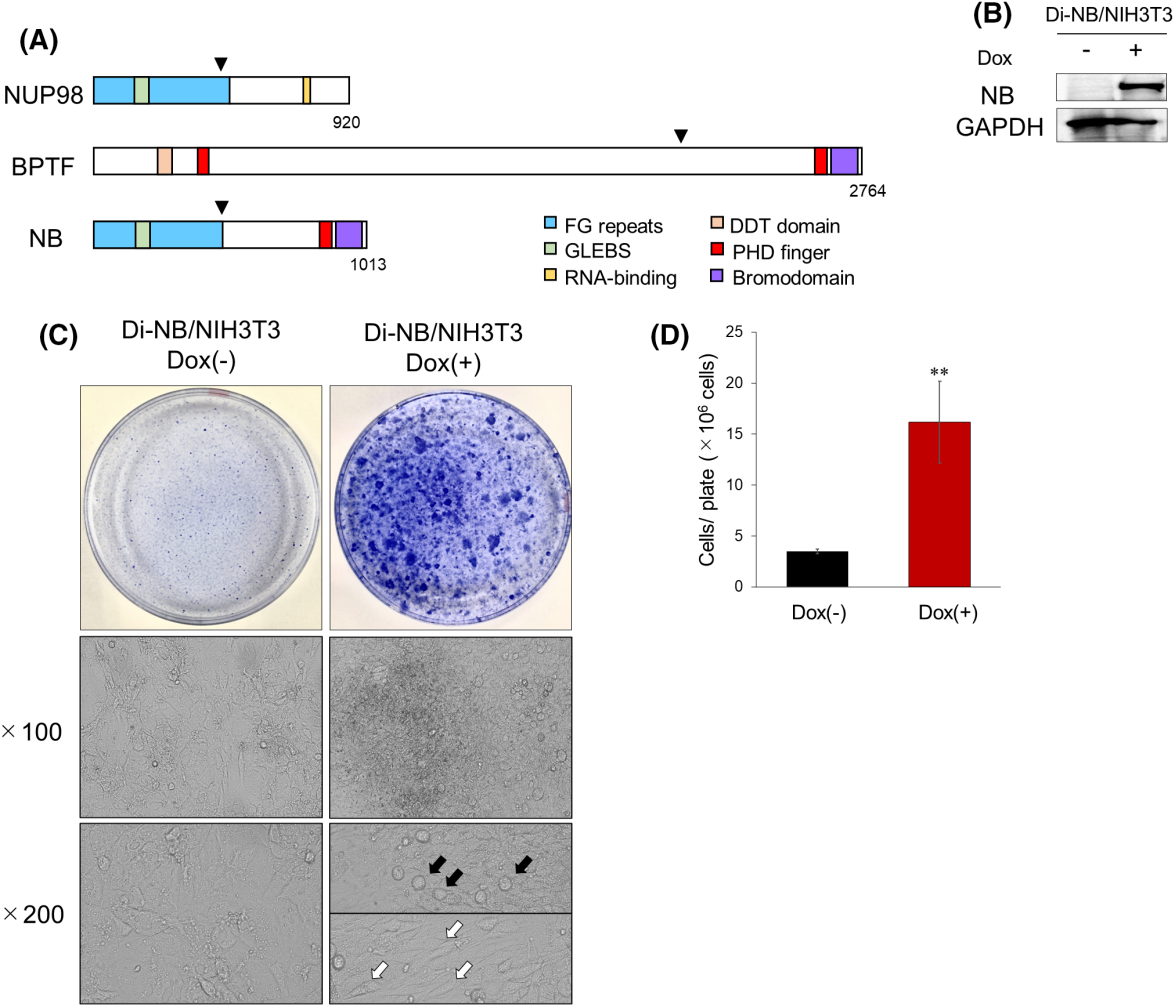
NUP98‐BPTF (NB) promoted NIH3T3 cell transformation. (A) Schematic representation of the structure of NB. The triangles represent fusion junctions. (B) Immunoblot analysis of NB in Di‐NB/NIH3T3. The cells were treated with or without 3 μM Dox for 72 h and then lysed for protein extraction. (C) Crystal violet staining of Di‐NB/NIH3T3 after 3 weeks of culture. Foci appeared approximately 2 weeks after the addition of Dox. The black and white arrows in the image indicate typical round or spindle‐shaped cells, respectively. (D) Cell numbers of Di‐NB/NIH3T3 after 3 weeks of culture. The cells were trypsinized and counted using the trypan blue exclusion method (*n* = 3). Data are presented as the mean ± SEM. **p < 0.01, by two‐tailed Student's *t‐*test.

NB consists of the amino‐terminal portion of NUP98, which includes two FG‐repeat domains, and the carboxy‐terminal portion of BPTF, which contains a PHD finger and bromodomain (Figure 1A). To examine whether NB induces cell transformation, we lentivirally transduced a Dox‐inducible NB expression vector into NIH3T3 cells and established Di‐NB/NIH3T3 (Figure S1). NB expression was induced by 3 μM Dox treatment in Di‐NB/NIH3T3 (Figure 1B). After 3 weeks of cell culture, NB‐expressing NIH3T3 cells formed multilayered foci, leading to the increased cell numbers (Figure 1C,D). Foci induced by exogenous NB expression contained round or spindle‐shaped cells (Figure 1C). These data showed that NB caused the oncogenic transformation of NIH3T3 cells.

### 3.2 NB upregulated *Pim1* expression in NIH3T3 cells

**FIGURE 2:**
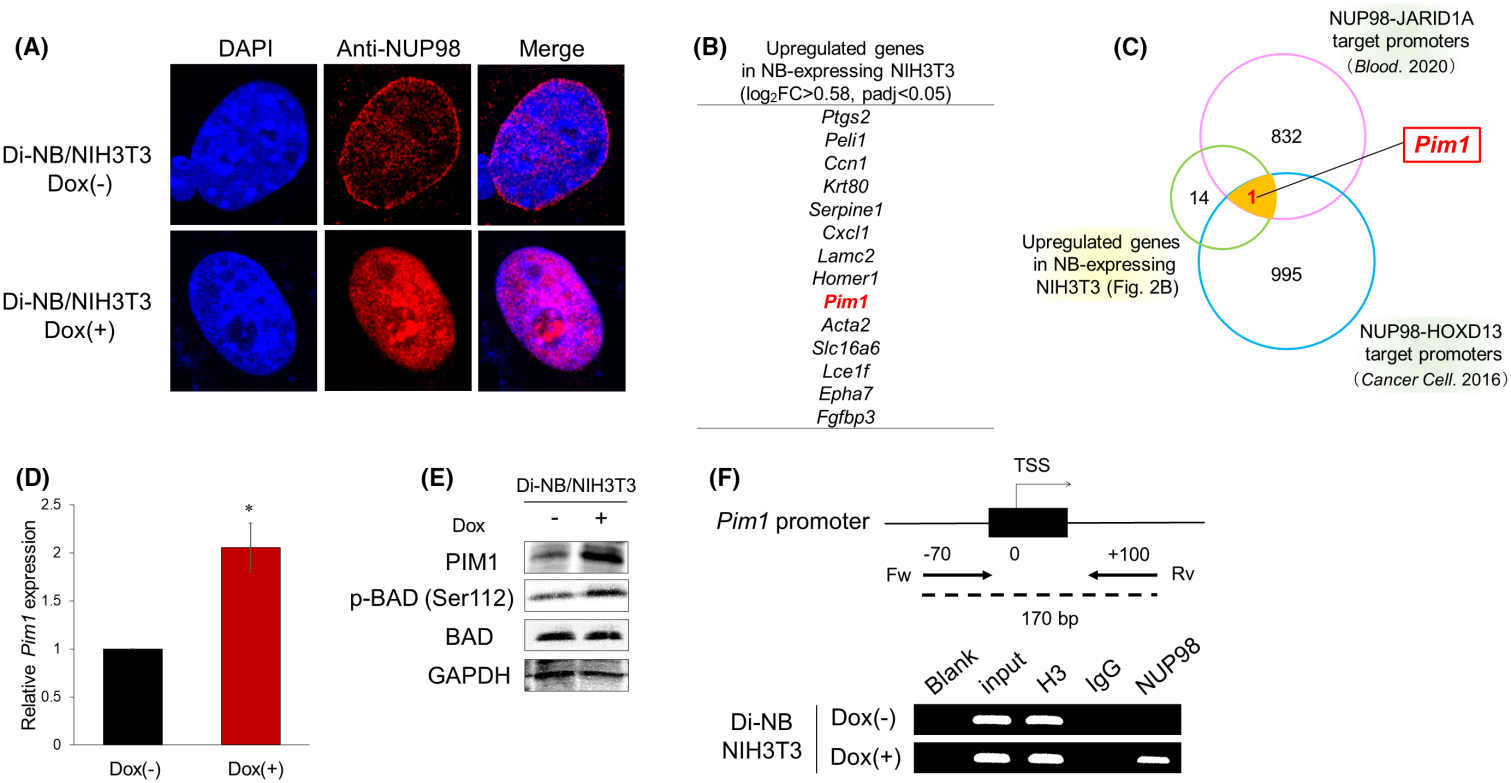
NB upregulated *Pim1* expression in NIH3T3 cells. (A) Subcellular localization of NB fusion protein in Di‐NB/NIH3T3. The cells were probed with an anti‐NUP98 antibody and then labeled with Alexa Fluor 568‐conjugated secondary antibody. The nuclei were counterstained with DAPI. (B) A list of 14 upregulated genes with log2 FC >0.58 (fold change>1.5), *p*_adj_<0.05, and base mean >50 in Dox‐treated Di‐NB/NIH3T3. (C) Venn diagram showing the overlap of the upregulated genes in NB‐expressing NIH3T3 cells and previously published target promoters of NUP98‐JARID1A and NUP98‐HOXD13. (D) Upregulation of *Pim1* in Dox‐treated Di‐NB/NIH3T3. The cells were treated with or without Dox for 72 h, and then total RNA was prepared and analyzed by RT‐qPCR. The values were normalized to the expression levels of *Gapdh* (*n* = 3). Data are presented as the mean ± SEM. *p < 0.05, by two‐tailed Student's *t‐*test. (E) Immunoblot analysis of PIM1, p‐BAD (Ser112), and BAD in Di‐NB/NIH3T3. The cells were treated as in Figure 1B. (F) NB bound to the *Pim1* promoter. The upper image shows the proximal regulatory region of *Pim1*. The lower image shows the results of the ChIP analysis of Di‐NB/NIH3T3. The cells were treated with or without Dox for 48 h to induce NB expression prior to the ChIP assay. Blank (distilled water only); input DNA; H3 (positive control), IgG (negative control), and NUP98 precipitated reactions were amplified with the indicated primer set.

Next, we investigated the subcellular localization of the NB fusion protein. Immunofluorescence showed that NB protein was predominantly located on the nucleus, while endogenous NUP98 was primarily localized to the NPC (Figure 2A), suggesting the possible involvement of NB in transcriptional regulation. To identify the key genes that are involved in NB‐mediated NIH3T3 cell transformation, we performed RNA‐seq and compared gene expression between Dox‐treated and Dox‐untreated Di‐NB/NIH3T3. We searched upregulated genes in Dox‐treated cells compared to untreated cells and extracted 14 genes satisfying the following criteria: log2 fold change >0.58 (fold change >1.5), adjusted *p*‐value <0.05, and base mean >50 (Figure 2B; Table S1). As some of the target genes of NB are expected to be common to those of other NUP98 fusion proteins, we searched for genes with target promoters of other NUP98 fusion proteins among the candidate genes. We reanalyzed the previously published ChIP‐seq datasets^9^, ^16^ and extracted genes with promoters bound by NUP98‐JARID1A and NUP98‐HOXD13 (Figure 2C). As shown in Figure 2C, *Pim1* was the only gene that was upregulated by NB and with a promoter bound by NUP98‐JARID1A and NUP98‐HOXD13. Therefore, we focused on *Pim1* as a potential target gene of the NB fusion protein. PIM1 is a highly conserved serine/threonine kinase that is constitutively active and is involved in cell transformation, proliferation, and survival through the phosphorylation of various downstream targets.^27^, ^28^ We performed RT‐qPCR analysis to examine the mRNA levels of *Pim1* in Di‐NB/NIH3T3. NB overexpression significantly upregulated *Pim1* expression in NIH3T3 cells (Figure 2D). Interestingly, NB did not induce the expression of other members of the Pim family, *Pim2*, and *Pim3* (Figure S2). Moreover, immunoblot analysis confirmed that the changes in the protein levels of PIM1 in NIH3T3 cells were consistent with the results of RT‐qPCR (Figure 2E). PIM1 promotes cell survival by phosphorylating the pro‐apoptotic protein BCL2‐associated agonist of cell death (BAD) at Ser112.^29^, ^30^ We confirmed the elevated phosphorylation of BAD at Ser112 in NB‐expressing NIH3T3 cells (Figure 2E), which may contribute to the survival of NIH3T3 cells at confluency.

Subsequently, we performed ChIP‐PCR using an anti‐NUP98 antibody to examine whether NB regulates *Pim1* transcription by binding to its promoter. As no study has identified the NUP98 fusion binding motif, we amplified the region within the *Pim1* promoter that was previously reported to be bound by NUP98‐JARID1A^16^ (−70 to 100 bp relative to the TSS). As shown in Figure 2F, NB bound to the *Pim1* promoter in the Dox‐treated Di‐NB/NIH3T3.

**FIGURE 3:**
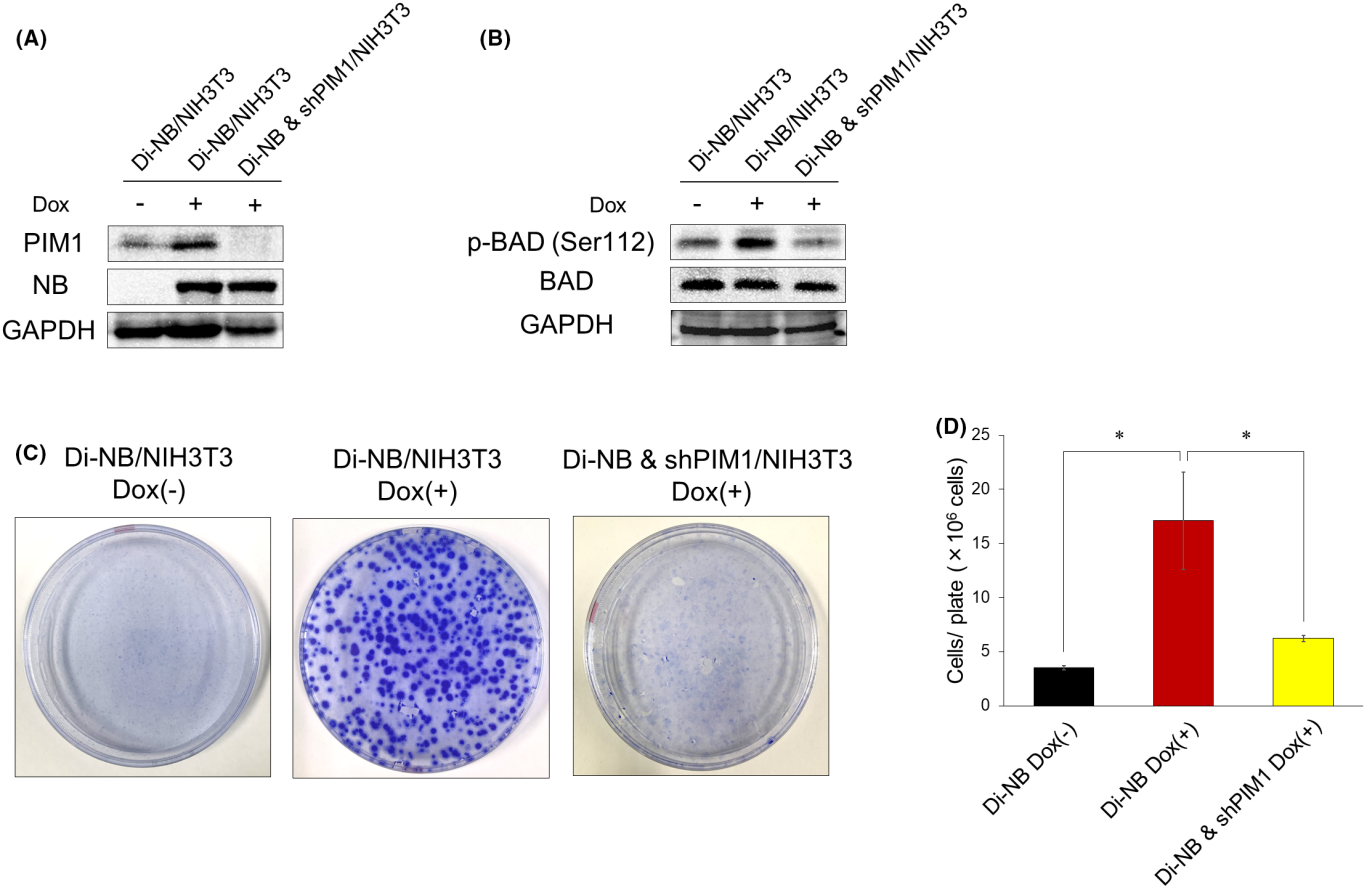
NB‐mediated NIH3T3 transformation is dependent on PIM1 expression. (A) Immunoblot analysis of PIM1 and NB after knockdown (KD) of PIM1 in Di‐NB /NIH3T3. The cells were treated as in Figure 1B. (B) Immunoblot analysis of p‐BAD (Ser112) and BAD after KD of PIM1 in Di‐NB /NIH3T3. The cells were treated as in Figure 1B. (C) Suppression of NB‐mediated NIH3T3 cell transformation by PIM1 KD. After 3 weeks of culture, the cells were stained with crystal violet to visualize the foci. (D) Reduced cell numbers of NB‐expressing NIH3T3 cells by PIM1 KD. The cells were counted as in Figure 1D. Data are presented as the mean ± SEM. *p < 0.05, by two‐tailed Student's *t‐*test.

To demonstrate that NB‐induced NIH3T3 transformation was mediated by PIM1, we constructed a Dox‐inducible *Pim1* shRNA expression vector and lentivirally transduced it into Di‐NB/NIH3T3 (Di‐NB & shPIM1/NIH3T3). Dox treatment induced NB expression and suppressed PIM1 expression in Di‐NB & shPIM1/NIH3T3 (Figure 3A). PIM1 knockdown decreased the NB‐induced phosphorylation of BAD (Figure 3B). Moreover, PIM1 knockdown suppressed NB‐induced NIH3T3 transformation and reduced cell numbers after 3 weeks of cell culture (Figure 3C,D). These results indicated that the NB‐mediated transformation of NIH3T3 cells was dependent on PIM1 expression.

### 3.3 Inhibition of the mTORC1 pathway by rapamycin suppressed NB‐induced NIH3T3 transformation

**FIGURE 4:**
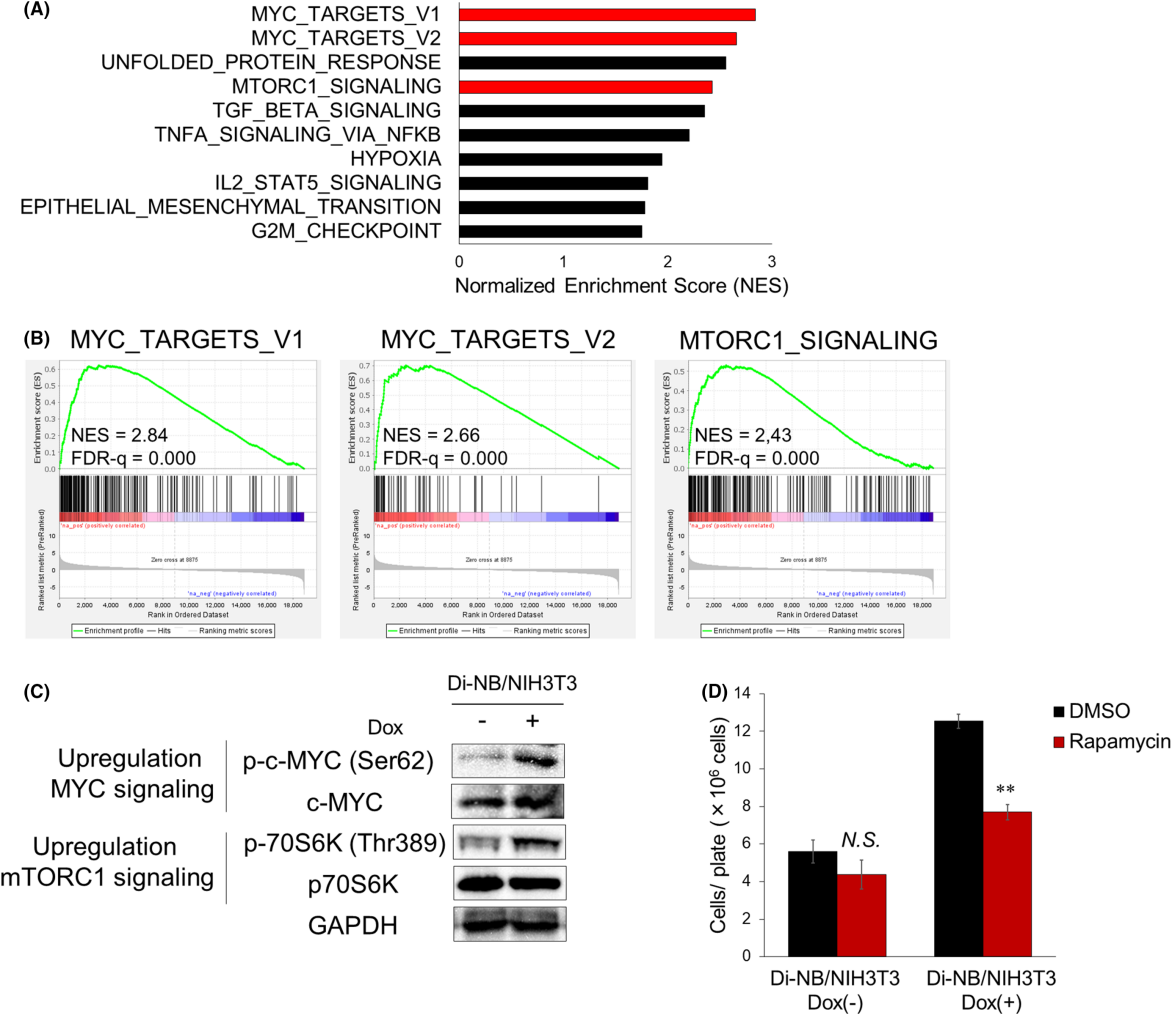
Inhibition of mTORC1 signaling by rapamycin suppressed NB‐mediated NIH3T3 cell transformation. (A) The top 10 enriched gene sets in upregulated genes in NB‐expressing NIH3T3 cells (NES >1.75). (B) MYC target genes and genes activated by mTORC1 signaling were enriched in upregulated genes in NB‐expressing NIH3T3 cells. (C) Elevated phosphorylation of c‐MYC and p70S6K in Dox‐treated Di‐NB/NIH3T3. The cells were treated with or without Dox for 72 h and then lysed for protein extraction. (D) Inhibition of NB‐mediated NIH3T3 cell transformation by rapamycin. The cells were cultured with or without 1 nM Rapamycin for 3 weeks. Dox and Rapamycin were added every other day. After 3 weeks of culture, the cells were counted as in Figure 1D. Data are presented as the mean ± SEM. **p < 0.01, by two‐tailed Student's *t‐*test.

Gene Set Enrichment Analysis using differentially expressed genes between the Dox‐treated and Dox‐untreated Di‐NB/NIH3T3 revealed that genes upregulated by exogenous NB expression were enriched for MYC target genes and downstream genes of mTORC1 signaling (Figure 4A,B; Table S2). Notably, PIM1 phosphorylates c‐MYC at Ser62 and stabilizes c‐MYC protein, which in turn increases the transcriptional activity of c‐MYC.^31^, ^32^ In addition, PIM1 activates mTORC1 signaling by directly phosphorylating PRAS40, an inhibitory subunit of the mTORC1 complex.^33^, ^34^ Therefore, the upregulation of these genes may be mediated through the phosphorylation of c‐MYC and p70S6K by PIM1 (Figure S3A). Immunoblotting analysis confirmed that NB increased the phosphorylation of c‐MYC and p70S6K in NIH3T3 cells (Figure 4C). These data suggested that NB activated MYC and mTORC1 signaling via the upregulation of PIM1.

We examined whether pharmacological inhibition of PIM1 inactivates MYC and mTORC1 signaling in NIH3T3 cells using TP‐3654, a second‐generation pan‐PIM inhibitor. We treated Di‐NB/NIH3T3 with Dox and various concentrations of TP‐3654 and found that 2 μM TP‐3654 reduced the phosphorylation of c‐MYC and p70S6K (Figure S3B). Although we tried to investigate whether inactivation of MYC and mTORC1 signaling by TP‐3654 can suppress the NB‐induced NIH3T3 cell transformation, we found it difficult because 2 μM TP‐3654 effectively suppressed the proliferation of the Dox‐untreated Di‐NB/NIH3T3 probably due to its nonspecific cytotoxicity (Figure S3C).

Rapamycin is a representative mTORC1 inhibitor, and several rapamycin derivatives have been FDA‐approved for the treatment of specific cancers.^35^ We investigated whether the inhibition of mTORC1 signaling by rapamycin suppressed NB‐mediated NIH3T3 transformation. We treated Di‐NB/NIH3T3 with Dox and various concentrations of rapamycin and confirmed that 1 nM rapamycin inhibited the phosphorylation of p70S6K without affecting its protein levels in NB‐expressing NIH3T3 cells (Figure S3D). Moreover, treatment with 1 nM rapamycin did not kill the Dox‐untreated Di‐NB/NIH3T3 cells (Figure S3E). The results of the focus formation assay showed that rapamycin significantly reduced the cell numbers of Dox‐treated Di‐NB/NIH3T3 but did not affect the growth of Dox‐untreated cells (Figure 4D). These results demonstrated the crucial role of mTORC1 signaling in NB‐driven tumorigenesis.

### 3.4 NB enhanced cell survival of a human T‐ALL cell line

**FIGURE 5:**
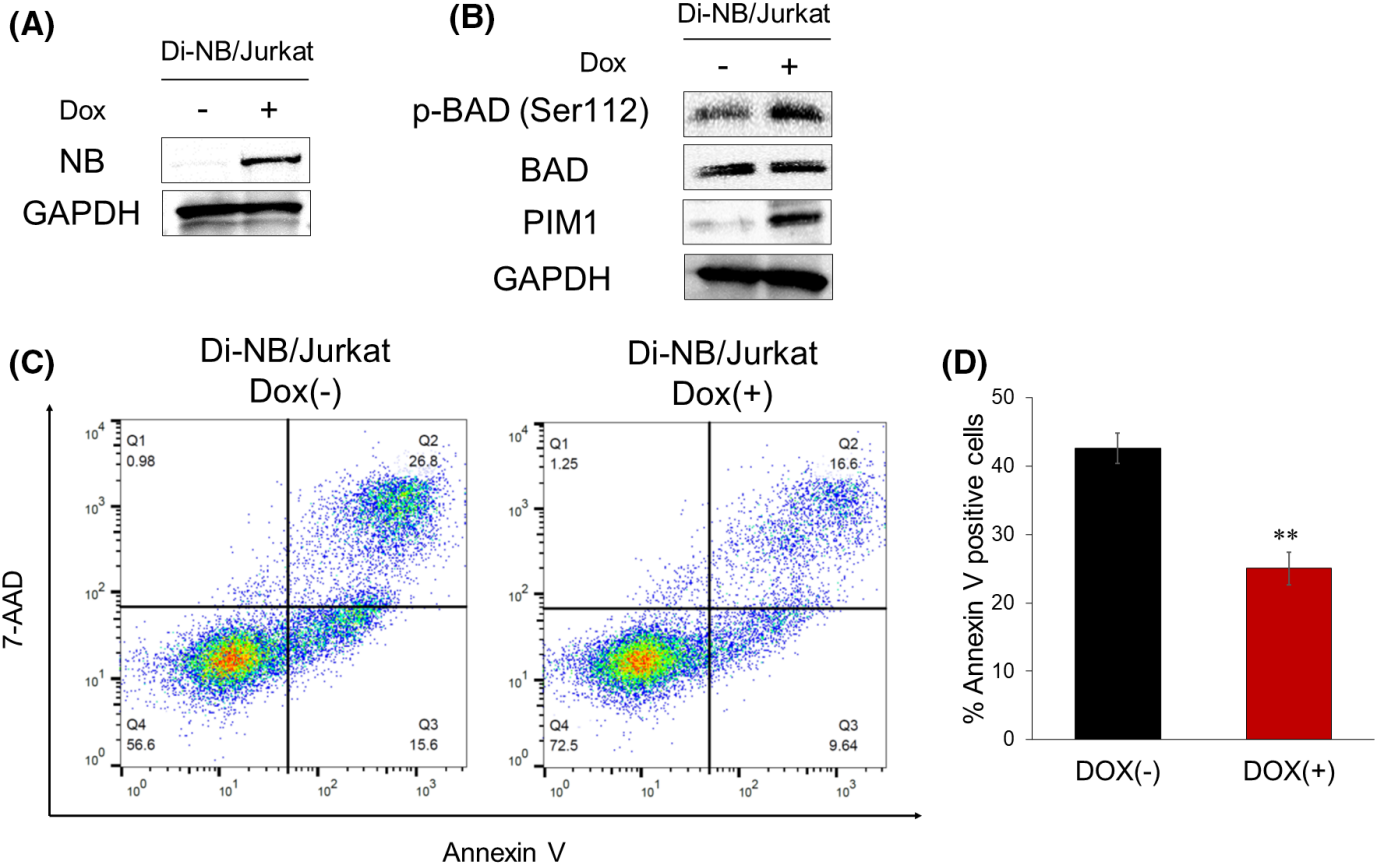
NB decreased serum starvation‐induced apoptosis of human T‐ALL cells. (A) Immunoblot analysis of NB in Di‐NB/Jurkat. Cells were treated as in Figure 1B. (B) Elevated BAD phosphorylation at Ser112 by NB in Di‐NB/Jurkat. The cells were serum‐starved in serum‐free RPMI 1640 medium with or without Dox for 48 h and then lysed for protein extraction. (C, D) Apoptotic cell death decreased by NB in Di‐NB/Jurkat. The cells were treated as in (B), and then the annexin V‐positive cells were scored by flow cytometric analysis (*n* = 3). Data are presented as the mean ± SEM. **p < 0.01, by two‐tailed Student's *t‐*test.

To clarify whether NB contributes to the development and maintenance of leukemia, we lentivirally transduced a Dox‐inducible NB expression vector into Jurkat human T‐ALL cells and established Di‐NB/Jurkat (Figure S4). NB expression was induced by 3 μM Dox treatment in Di‐NB/Jurkat (Figure 5A). Exogenous NB expression did not affect the proliferation of Jurkat cells (data not shown), probably because Jurkat cells originally harbor multiple genetic abnormalities.^36^ To examine whether NB promotes the survival of leukemic cells, Di‐NB/Jurkat were subjected to serum deprivation. NB increased PIM1 expression and phosphorylation of BAD at Ser112 in serum‐starved Di‐NB/Jurkat (Figure 5B). Moreover, apoptotic cell death induced by serum deprivation was significantly decreased by exogenous NB expression (Figure 5C,D). Collectively, our results showed that NB enhanced the survival of T‐ALL cells by increasing PIM1 expression and phosphorylation of BAD.

## 4 DISCUSSION

This study uncovered the important roles of NB in the oncogenic process. Our results showed that NB promoted cell transformation and survival by upregulating PIM1 expression. NUP98 with distinct fusion partners share several common target genes, which are essential for leukemogenesis. Many NUP98 fusion proteins, including NUP98‐HOXA9, NUP98‐HOXD13, NUP98‐NSD1, and NUP98‐JARID1A, are associated with the overexpression of *HOXA* cluster genes, leading to leukemic transformation.^9^, ^10^, ^37^ However, NB did not upregulate *HOXA* genes in our NIH3T3 model (Table S1). We speculate that this is because NIH3T3 is a nonhematopoietic cell line. Because the regulatory mechanism of gene expression highly depends on cell type, the effects of NB observed in NIH3T3 cells need to be examined using human leukemia cell lines or primary murine hematopoietic cells.

In a previous study, microarray analysis revealed that NUP98‐HOXA9 upregulates *PIM1* expression in human CD34^+^ cells.^38^ In addition, we reanalyzed the published ChIP‐seq data and found that *PIM1* was a common promoter target of NUP98‐JARID1A and NUP98‐HOXD13 (Figure 2C). These data suggest that *PIM1* upregulation is a common phenomenon in some cases of NUP98 fusion‐positive leukemia. We further confirmed that NB bound to the *Pim1* promoter in NIH3T3 cells (Figure 2F). Because both NUP98 and BPTF lack a DNA‐binding domain, it is reasonable to speculate that NB interacts with other adaptor proteins to associate with the *PIM1* promoter. For instance, the components of the NSL/MLL1 complex are candidate interaction partners of NB because NUP98 fusions, such as NUP98‐HOXA9 and NUP98‐HOXD13, interact with the NSL/MLL1 complex and promote leukemogenesis.^9^ Further experiments are required to identify the NB‐interacting proteins at the *PIM1* promoter region.

Previous research revealed that mutation of the PHD domain of BPTF abolished the NB‐induced transformation of murine hematopoietic progenitor cells,^39^ suggesting that the BPTF portion is also critical for the oncogenic transformation. However, the functions of the BPTF portion in transcriptional regulation remain unclear. Wild‐type BPTF regulates chromatin remodeling and activates transcription by binding to H3K4me3 via the PHD domain. Because the BPTF portion of NB retains the PHD domain, it may serve as a transcriptional activator of genes with a promoter bound by the NUP98 moiety. Detailed studies should be conducted to clarify whether the interaction of the PHD finger with H3K4me3 is necessary for the transcriptional regulation of *PIM1* by NB fusion.

Elevated expression levels of *PIM1* have been found in various hematopoietic malignancies, and its expression is associated with poor prognosis.^40^ Several studies have shown that PIM1 may serve as a potential therapeutic target in T‐ALL.^41^, ^42^, ^43^, ^44^, ^45^ Although direct inhibition of NB is challenging, targeting its downstream pathways is a promising therapeutic strategy. We found that MYC and mTORC1 signaling were significantly activated in NB‐expressing NIH3T3 cells (Figure 4A,B). Although pharmacological inhibition of c‐MYC is difficult,^46^ rapamycin is widely utilized as a specific mTORC1 inhibitor.^35^ The inhibition of mTORC1 signaling by rapamycin suppressed NB‐mediated NIH3T3 cell transformation (Figure 4D). These results indicate that rapamycin is a promising compound that controls oncogenic signaling activated by NB fusion.

Although we showed the effect of exogenous NB expression in NIH3T3 and Jurkat cells, we failed to consider the loss of one normal copy of NUP98 and its splicing variant, NUP96. The loss of NUP98‐96 by NUP98 translocations deregulates cell cycle control and promotes tumorigenesis in the presence of additional events that suppress cell death.^47^ In the present study, NB suppressed apoptosis by upregulation of PIM1 and phosphorylation of BAD (Figure 5). Therefore, NB may promote tumorigenesis by disrupting the NUP98‐96 locus and suppressing apoptosis.

In summary, we demonstrated the oncogenic transformation ability of NB and identified PIM1 as a critical target in NB‐positive leukemia. Further studies are required to examine the effect of NB on lymphoid transformation and its leukemogenic potential in vivo.

## AUTHOR CONTRIBUTIONS

**Mina Noura:** Conceptualization (lead); formal analysis (lead); funding acquisition (equal); investigation (lead); project administration (lead); writing – original draft (lead). **Sakura Tomita:** Investigation (supporting). **Takahiko Yasuda:** Data curation (lead); formal analysis (equal). **Shinobu Tsuzuki:** Writing – review and editing (equal). **Hitoshi Kiyoi:** Writing – review and editing (equal). **Fumihiko Hayakawa:** Funding acquisition (equal); supervision (equal); validation (equal); writing – review and editing (equal).

## FUNDING INFORMATION

This work was supported by JSPS KAKENHI Grant Numbers 21 K19505, 22H03102 (to F.H.), and 23 K15298 (to M.N.); and Grants for Practical Research for Innovative Cancer Control Grant Numbers JP21ck0106607 and JP23ck0106851 (to F.H.) from the Japan Agency for Medical Research and Development (AMED).

## CONFLICT OF INTEREST STATEMENT

H.K. received research funds from FUJIFILM, Kyowa Hakko Kirin, Otsuka, Perseus Proteomics, Daiichi Sankyo, AbbVie, CURED, Astellas Pharma, and Bristol‐Myers Squibb; scholarship funds from Zenyaku Kogyo, Nippon Shinyaku, Chugai, Astellas Pharma, Kyowa Hakko Kirin, Takeda, Sumitomo Dainippon Pharma, Sanofi, Eisai, and Ono; and honoraria from AbbVie, Chugai, Astellas Pharma, and Novartis. The other authors declare no conflicts of interest.

## ETHICS STATEMENT

Approval of the research protocol by an Institutional Reviewer Board: N/A.

## Supporting information


Table S1.

Table S2.

Figure S1.
Figure S2.
Figure S3.
Figure S4.

## Data Availability

The data that support the findings of this study are available from the corresponding author upon reasonable request.
