# Supplementary material for: NUP98‐BPTF promotes oncogenic transformation through PIM1 upregulation
Source: Cancer Med. 2024 Jun 28;13(13):e7445. doi: 10.1002/cam4.7445 (PMC11212001; doi:10.1002/cam4.7445)
Supplement: Supplementary file 3 — Figure S1. Figure S2. Figure S3. Figure S4. [file CAM4-13-e7445-s001.docx]

**Supplemental Figures**

**NUP98-BPTF promotes oncogenic transformation through PIM1 upregulation**

Mina Noura, Sakura Tomita, Takahiko Yasuda, Shinobu Tsuzuki, Hitoshi Kiyoi, Fumihiko Hayakawa.

**
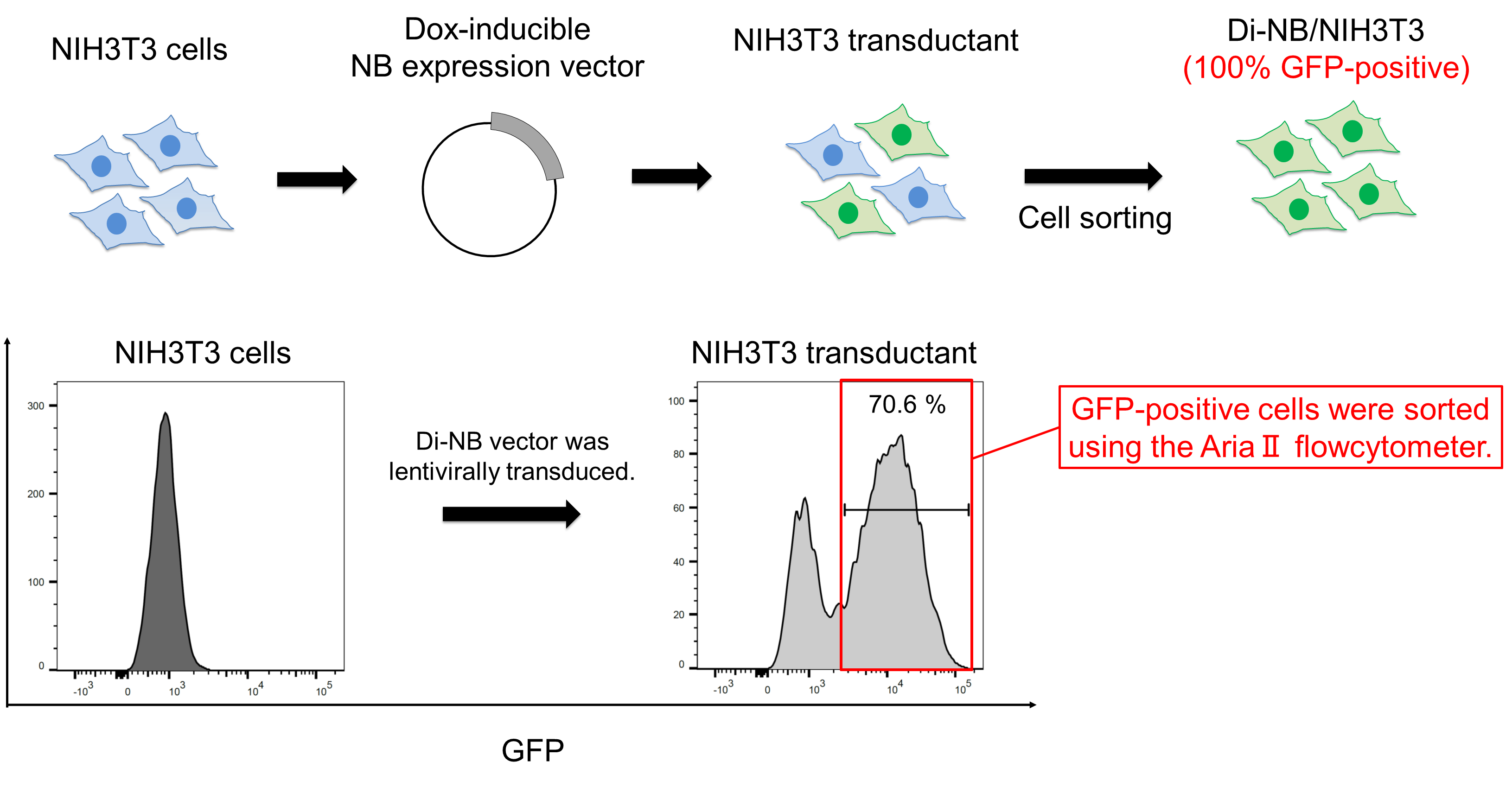
**

**Supplemental Figure 1**

Flow cytometric sorting of GFP-positive NIH3T3 cells. The NIH3T3 cells were lentivirally transduced with a Dox-inducible NB expression vector. Successfully transduced GFP-positive cells were sorted using a flow cytometer (Di-NB/NIH3T3).


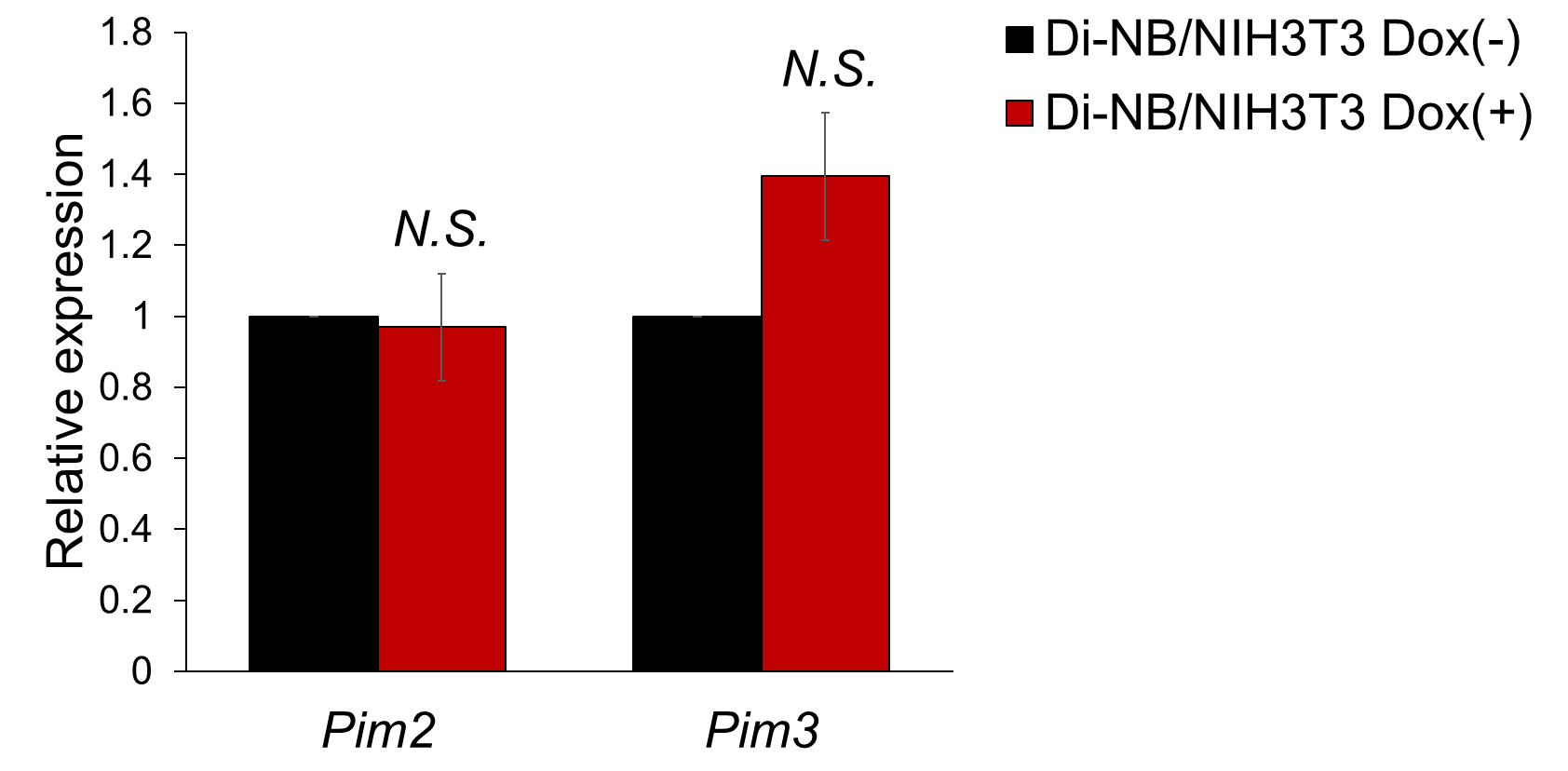


**Supplemental Figure 2**

Exogenous NB expression did not change the mRNA expression levels of *Pim2* and *Pim3*. The cells were treated with or without Dox for 72 h, and then total RNA was extracted and analyzed by RT-qPCR. The values were normalized to the expression levels of *Gapdh* (n = 3).

**
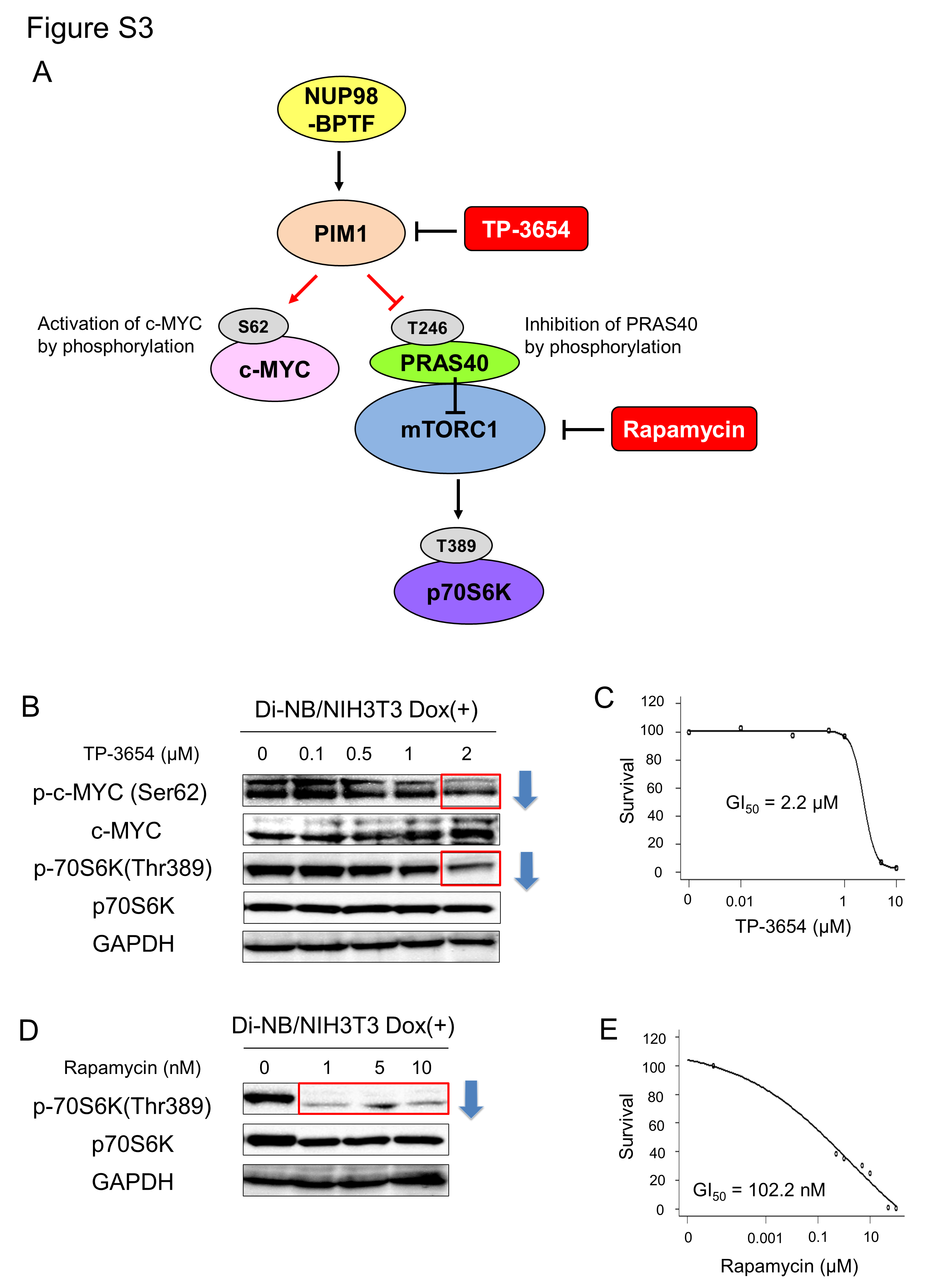
**

**Supplemental Figure 3**

(A) Schematic illustration of MYC and mTORC1 signaling activated by NB-induced PIM1 expression.

(B) TP-3654 decreased the phosphorylation levels of c-MYC and p70S6K in Dox-treated Di-NB/NIH3T3. The cells were treated with Dox and the indicated concentrations of Rapamycin for 72 h and then lysed for protein extraction.

(C) Dose-response curves of TP-3654 in Dox-untreated Di-NB/NIH3T3. The cells were treated with various concentrations of TP-3654 for 72 h. GI_50_ values of TP-3654 were calculated (n = 3).

(D) Rapamycin decreased the phosphorylation levels of p70S6K in Dox-treated Di-NB/NIH3T3. The cells were treated with Dox and the indicated concentrations of rapamycin for 72 h and then lysed for protein extraction.

(E) Dose-response curves of rapamycin in Dox-untreated Di-NB/NIH3T3. The cells were treated with various concentrations of rapamycin for 72 h. GI_50_ values of Rapamycin were calculated (n = 3).

**
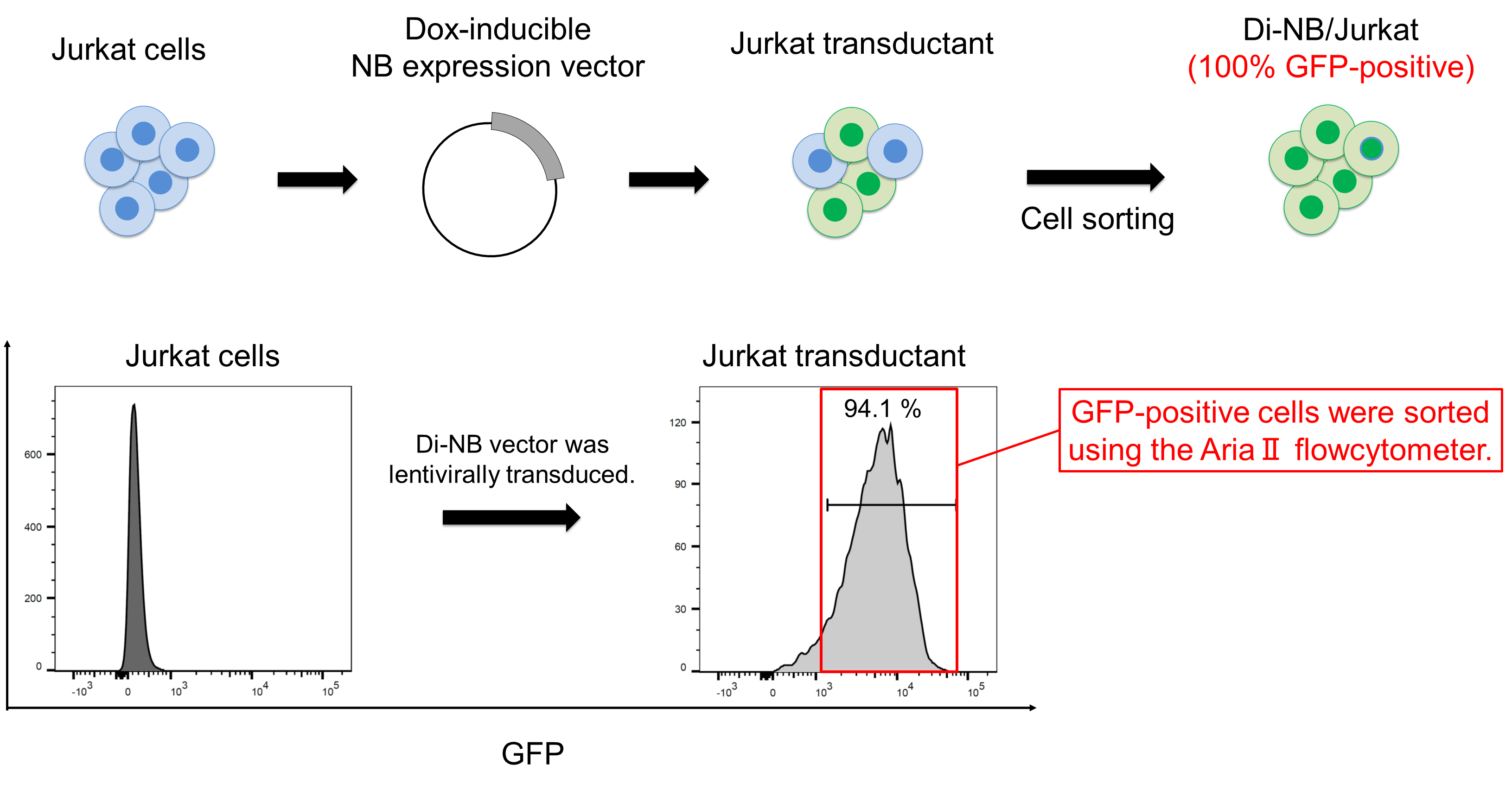
**

**Supplemental Figure 4**

Flow cytometric sorting of GFP-positive Jurkat cells. Jurkat cells were lentivirally transduced with a Dox-inducible NB expression vector. Successfully transduced GFP-positive cells were sorted using a flow cytometer (Di-NB/Jurkat).
